# Continuous Measurement of Reconnaissance Marines in Training With Custom Smartphone App and Watch: Observational Cohort Study

**DOI:** 10.2196/14116

**Published:** 2020-06-15

**Authors:** Leslie Saxon, Brooks DiPaula, Glenn R Fox, Rebecca Ebert, Josiah Duhaime, Luciano Nocera, Luan Tran, Mona Sobhani

**Affiliations:** 1 University of Southern California Center for Body Computing Keck School of Medicine Playa Vista, CA United States; 2 University of Southern California Marshall School of Business Los Angeles, CA United States; 3 United States Marine Corps Reconnaissance Training Company Camp Pendleton, CA United States; 4 University of Southern California Department of Computer Science Viterbi School of Engineering Los Angeles, CA United States

**Keywords:** military, marines, wearable devices, wearable technology, smartphone, mobile app

## Abstract

**Background:**

Specialized training for elite US military units is associated with high attrition due to intense psychological and physical demands. The need to graduate more service members without degrading performance standards necessitates the identification of factors to predict success or failure in targeted training interventions.

**Objective:**

The aim of this study was to continuously quantify the mental and physical status of trainees of an elite military unit to identify novel predictors of success in training.

**Methods:**

A total of 3 consecutive classes of a specialized training course were provided with an Apple iPhone, Watch, and specially designed mobile app. Baseline personality assessments and continuous daily measures of mental status, physical pain, heart rate, activity, sleep, hydration, and nutrition were collected from the app and Watch data.

**Results:**

A total of 115 trainees enrolled and completed the study (100% male; age: mean 22 years, SD 4 years) and 64 (55.7%) successfully graduated. Most training withdrawals (27/115, 23.5%) occurred by day 7 (mean 5.5 days, SD 3.4 days; range 1-22 days). Extraversion, positive affect personality traits, and daily psychological profiles were associated with course completion; key psychological factors could predict withdrawals 1-2 days in advance (*P*=.009).

**Conclusions:**

Gathering accurate and continuous mental and physical status data during elite military training is possible with early predictors of withdrawal providing an opportunity for intervention.

## Introduction

### Background

Owing to the nature of the US conflicts in Iraq and Afghanistan, which span nearly two decades, the US military comprised many experienced warfighters who are highly trained and specialized [1,2]. Service members volunteer for training and selection to serve in elite and specialized US military units. One such group, Reconnaissance (Recon) Marines, undergoes intense and specialized psychological and physical training that is associated with high attrition rates, most of which occurs in the initial phase of training, and as a result, only approximately half are able to complete the nearly 90-day initial training course [3]. There is widespread acknowledgment that those who complete the course are highly adept warfighters, many of whom will serve for one to two decades.

Owing to continued global threats, there is a need to graduate more highly trained Recon Marines, without degrading performance standards [4,5]. Yet, as Recon training is exceedingly rigorous, the majority of the training failures are trainee-initiated, commonly referred to as *Drop on Request* (DOR) [3]. It is not known which mental and physical factors are predictors of success or failure in training. Some previous studies have used existing military assessments to investigate factors that best predict success or failure [3], whereas others have incorporated wearable sensors to collect and analyze physiologic signals [6,7] or analyzed personality and psychological variables as predictors [8-12]. However, none of these studies have analyzed existing military assessments along with continuously collected physiologic, personality, and psychological data to predict success or failure in military trainings. Furthermore, no studies have collected longitudinal and continuous physiological signals, and few studies have been conducted in naturalistic military training scenarios without disrupting standard procedures.

### Objectives

To help determine novel predictors of success or failure in training, we collected continuous mental and physical status of Recon trainees to build comprehensive models capable of identifying the collective load of Recon training and the thresholds at which trainees are most likely to remove themselves from the course. In doing so, novel insight into the mental and physical processes associated with these factors can inform the theories of human performance and improve desired outcomes in elite military training.

The University of Southern California, Center for Body Computing, a digital health and human performance research center, partnered with Reconnaissance Training Company leadership at Camp Pendleton, California, in 2016 to design and implement a human performance study aimed at obtaining continuous assessments of Recon trainees, using connected technology to garner insights and identify novel predictors of training completion. Over the course of 36 months, requirements were identified that included the need to collect (1) personality profiles, as prior military studies had identified personality traits such as grit to be predictive of training success [4,13,14]; (2) physiologic data including heart rate and work output (measured via accelerometer and photoplethysmography activity and calorie expenditure) during training exercises taking place in both land and water; (3) daily assessment of sleep, nutrition, and hydration; and (4) daily assessment of mental and physical pain and attitudes regarding successful completion of training. Our collective goal was to understand whether this new information could have additive value to standard measures of performance already being collected as well as be used to confirm or refute subjective instructor evaluation of trainee status. Another critical concern was to develop a study protocol that collected these continuous measures without disrupting the culture and cadence of training. We also identified the need to develop specialized data collection processes, sensors, and software that would engage and achieve adherence from the trainees throughout the course.

## Methods

### Study Subjects

**Figure 1:**
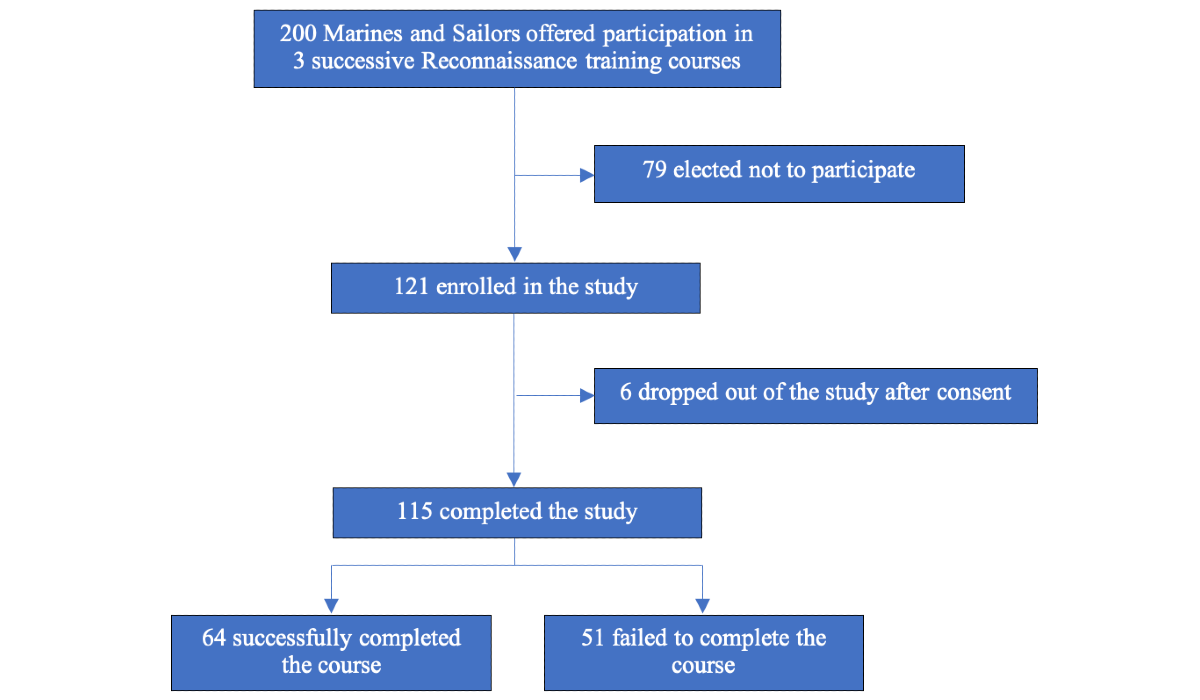
Marine and sailor study enrollment description and completion.

The 3 successive classes of Marines and Sailors entering the 25-day Basic Reconnaissance Primer Course (BRPC) of the Reconnaissance Training Company at Camp Pendleton, California, were offered study enrollment (April 26, 2018, to August 17, 2018) on the first day of orientation (Figure 1). A total of 60.5% (121/200) of the trainees consented to participate. Study personnel explained that the study was completely voluntary and that the study data would not be shared with course instructors. Of the 121 who consented, a total of 6 (5.0%) trainees dropped out of the study, and we did not collect data on their reasons for withdrawal. The study was approved by the Institutional Review Boards of the University of Southern California and Marine Corps Training and Education Command (University of Southern California IRB HS-17-00729 and Training and Education Command Human Subject Research Approval DoDI 3216.16D). Sample size was determined by the number of trainees willing to participate in the study. Informed consent was obtained using the mobile app. Baseline demographics of the study subjects are summarized in Table 1.

**Table 1:** Baseline demographics of study subjects.

Characteristic		Value
Age (years), mean (SD)		22 (4)
Height (cm), mean (SD)		175 (8)
Weight (kg), mean (SD)		77 (9)
Participated in high school sports (N=104), n (%)		79 (76.0)
Marital status: single (N=115), n (%)		107 (93.0)
Time in military (months), mean (SD)		23 (23)
**Military rank (N=115), n (%)**		
	Marine officer	4 (3.5)
	Marine enlisted	105 (91.3)
	Navy enlisted	12 (10.4)

### Basic Reconnaissance Primer Course Measures

The 25-day BRPC is the initial phase of Reconnaissance training that must be successfully completed to advance to the 60-day Basic Reconnaissance Course (BRC), which is required for graduation. Marines and Sailors are accepted into the BRPC from other marine operational specialties or after enlistment and completion of entry-level infantry training.

**Figure 2:**
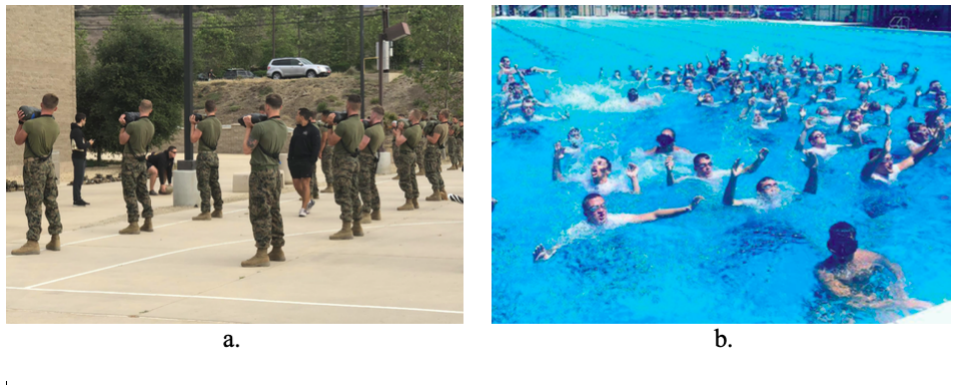
Photographs of trainees engaged in land exercises (a) and water exercises (b).

The BRPC consists of structured daily training on land and water that embeds the cognitive and physical assessments that trainees have to complete, benchmarked to time or quality standards. The aquatics training includes various high-intensity underwater and treading drills that impose intense psychological and physical stressors. This course selectively screens and prepares trainees to acquire the mental and physical skills taught in BRC, such as land navigation, coastal piloting, and radio communications, which render them able to serve as a Reconnaissance Marine within the Fleet Marine Forces (Figure 2).

### Reasons for Training Failures

Trainees can drop out of training by choice on request (DOR), and medical personnel can remove trainees from the course if they have an illness or injury (medical) or instructors determine that trainees pose a safety hazard to themselves or other trainees (safety) or fail to meet one or more of the course training standards (performance). Trainees that DOR are not permitted to repeat the training course and are then assigned back to their prior unit or are assigned to a different training school to obtain another military occupational specialty. Those who are excluded from the course for medical, safety, or performance reasons may repeat the course at the next scheduled course initiation.

### Study Software and Hardware

**Figure 3:**
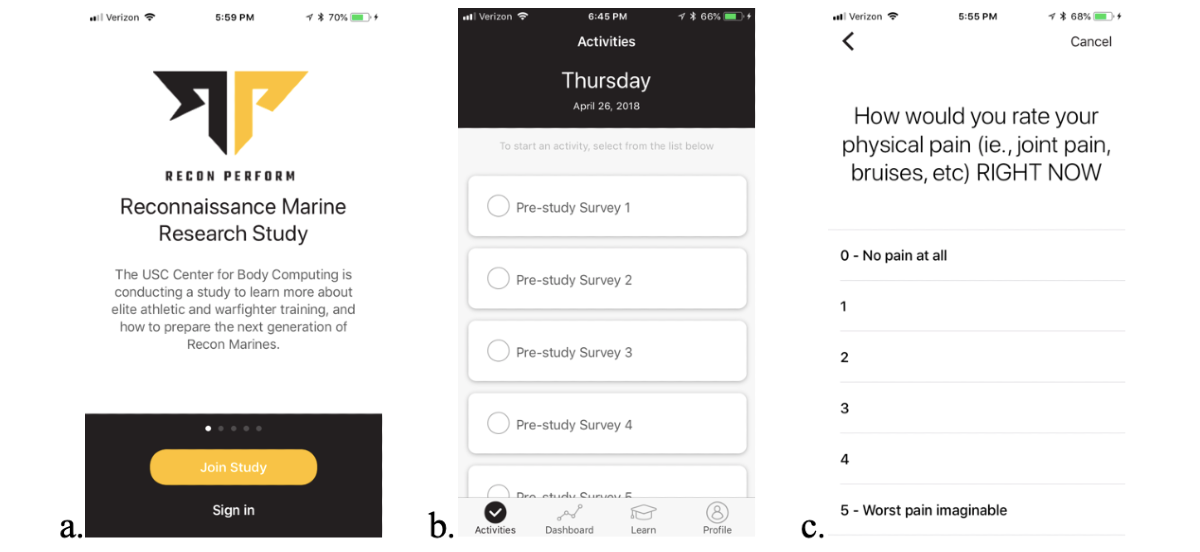
Sample study app screenshots: (a) initial study screen, (b) prestudy survey screen, and (c) daily survey.

A specially designed software app, used for subject consent and data collection, was designed on Apple’s ResearchKit platform (Apple Inc and Thread Research) for the purposes of this study (Figure 3) [15-17]. At the time of consent, iPhones and Apple Watches were distributed to study subjects, and they received a structured tutorial on how to use the app and Watch and were instructed to wear the Watch for collection all day and all night. We expected that attrition due to compliance and user error could be a substantial hurdle to gathering complete datasets, as the paradigm required subjects to use the Apple Watch to log in their workouts via workout mode before each activity, which allowed for stronger data collection but caused a need for the battery to be recharged every few hours. To increase compliance, study personnel supported Watch and iPhone battery charging during training days at scheduled breaks and redistributed them to trainees up to 4 times during a 24-hour cycle to increase compliance. Study personnel also worked with instructors to ensure that all subjects had their devices before beginning the class. During high-intensity training drills, such as land-based or aquatic training, Watches were put in the workout mode to collect data with a higher sampling rate (sampling heart rate continuously every 5 seconds vs the standard sampling frequency of every 5 min) [18,19].

### Study Measures

Table 2 lists all study metrics that were collected. A range of validated surveys were administered at the beginning of the study that assessed various personal characteristics, such as personality type, emotional processing, outlooks on life, and mindfulness. To assess personality, we used the Big Five Inventory (BFI), which is a well-validated personality construct that has been previously used in military human performance and leadership research [8,11,20-23]. We also included the Psychopathic Personality Inventory-Revised to capture additional personality traits [24]. As positive affect has been associated with sport performance [25] and resilience [26], we included the Positive and Negative Affect Scale (PANAS) [27]. To assess outlook on life and mindfulness, we used the Satisfaction with Life Scale (SWLS) [28] and Five Facet Mindfulness Questionnaire (FFMQ) [29], respectively. As grit and resilience have been identified as important traits for success in intense military trainings from previous research [12,13,15], we included the Grit Scale [12,13] and the Ego Resilience Scale (ERS) [30]. The mobile app prompted subjects to answer daily surveys after completion of training and before going to sleep, which rated emotional and physical pain, well-being, confidence in course completion and instructor support, hydration, nutrition, and sleep status (Multimedia Appendix 1). The Apple Watch collected heart rate and activity measures [18,19].

**Table 2:** Study metrics by collection method.

Method and metric			Measurement
**iPhone app**			
	**Consent**		
		Questionnaires: prestudy	Demographic survey Big Five Inventory [20-22] Psychopathic Personality Inventory-Revised [24] Positive and Negative Affect Schedule [27] Satisfaction with Life Scale [28] Five Facet Mindfulness Questionnaire [29] Grit Scale [12,13] The Ego Resilience Scale [30]
		Daily questionnaires (Multimedia Appendix 1)	Mental and physical pain scale (1-5) Sleep, hydration, and nutrition (self-report) Confidence in instructors and graduation (4 questions)
**Apple Watch**			
	**Heart rate (beats per minute)**		
		Daily activity	Steps Resting and active energy expenditure (calories)

### Statistical Plan

Statistical analyses were performed using R Statistical Software (Bell Laboratories) [31]. Percentages were calculated for course completion categories. For the DOR withdrawal requests, the time of day and nearest training event were analyzed for patterns. To examine whether there was an association between course completion categorical variable and students’ age, weight, height, and years in the Marine Corps, one-way analysis of variance was performed. A chi-square test of goodness-of-fit was performed to assess whether there was an equal distribution of rates of marital status, military rank, number of high school sports played, and number of previous BRPC attempts across course completion categories [32]. Normality of data was assessed using Kolmogorov-Smirnov tests [33]. The differences between course completion categories on physical test standard assessments were assessed using Wilcoxon rank sum tests [34], with significance set at *P*<.05, two-tailed. The 3-mile hike was performed by only 2 cohorts, and the distribution was as follows: Pass (ie, successful course completion), n=46; DOR, n=6; medical, n=4; safety, n=6; and performance, n=4. Descriptive statistics for the Apple Watch daily metrics were calculated for each individual using the number of days in the course. The Watch metrics were visually inspected for outliers using histograms of all raw scores for each subject across all workouts. Values of zero (<0.0001% of all values) were removed from the analyses because they could skew data and likely resulted from the Watch not being worn. The R outlier package was used to identify if zero values were sufficiently outside the distribution. Wilcoxon rank sum tests were used to assess differences between groups on Apple Watch metrics including heart rate, daily steps, active energy, and resting energy as well as daily survey assessments. To investigate the factors that may specifically contribute to voluntary course withdrawal (DORs), we compared the DOR and Pass groups directly for certain metrics, including baseline personality assessments and daily survey assessments. A random forest classifier was also used to classify course completion status from baseline personality surveys [35,36]. Wilcoxon rank sum tests were used to assess differences between DOR and Pass groups’ mean ratings for daily survey assessments on a day-to-day basis.

## Results

### Course Completion

The summary data for the course completion of all 3 BRPC study subjects are shown in Multimedia Appendix 2. Of the 115 subjects who completed the study, a total of 64 (55.7%) trainees successfully completed the course, and the reasons for failure to complete were DORs (27/115, 23.5%), medical (10/115, 8.7%), safety (8/115, 7.0%), and performance (6/115, 5.2%). The majority of training failures were trainee-initiated DORs, and these occurred, with one exception, by day 7 (mean 5.5 days, SD 4 days; range 1-22 days). There were no significant differences in course completion rates between successive classes (X^2^_2_=15.1; *P*>.05).

The timing and context of DORs fell into a consistent pattern, and 93% of drops occurred before an impending aquatic event or in the training pool. The DORs that did not occur before an impending aquatic event were observed at the beginning of the training day in a waiting area outside of the training schoolhouse. There were no significant differences between course completion groups with respect to age, height, weight, history of high school sports participation, marital status, years in the Marine Corps, Marine rank, or previous BRPC attempts (*P*>.05).

### Course Training Standards and Course Completion

**Figure 4:**
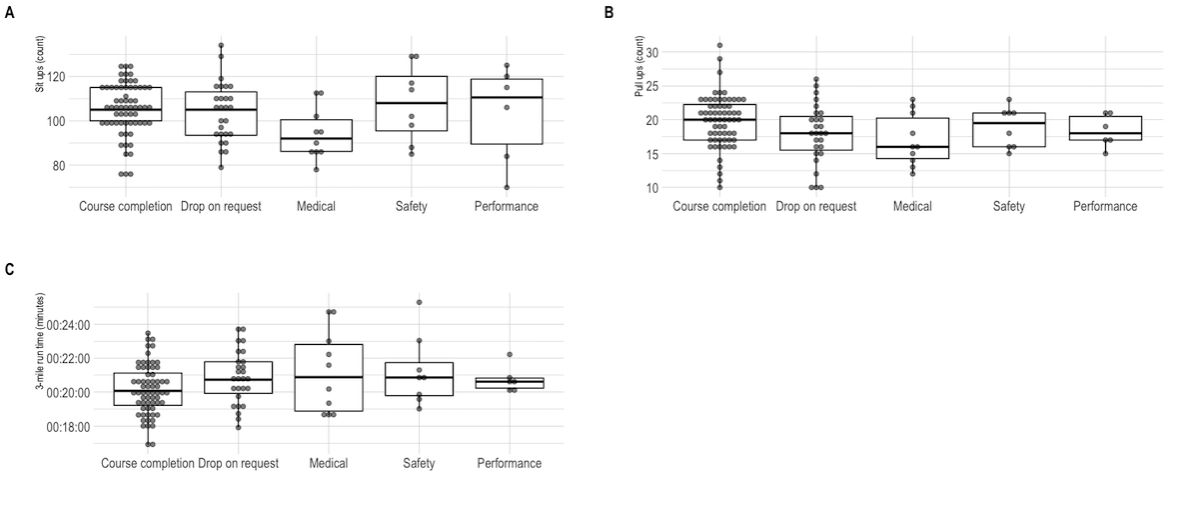
Course completion results for test standard assessments of (a) sit-ups; (b) pull-ups, timed; and (c) 3-mile run.

Figure 4 shows the course completion results of all study subjects according to the physical test standard assessments of sit-ups, pull-ups, and a timed 3-mile run. The median number of sit-ups performed was not significantly different between the Marines who completed the course and each category of failure (*P*>.05). The number of pull-ups was significantly lower in the DOR group (*P*=.049) and in the group that failed for medical reasons (*P*<.05) compared with the group who completed the course. The time on the 3-mile run standard trended toward a significant difference between the group that requested a DOR compared with those that passed (*P*=.05). In addition, there was no significant difference between the median 3-mile hike time and the reason for training withdrawal (*P*>.05).

### Physiologic Apple Watch Data and Course Completion

Table 3 provides a summary of 24-hour heart rate data and day and night heart rate data for the subject cohort over the 25-day course, according to course completion categories. Outlier analyses revealed no outliers in the data. The range values displayed in the table indicate that at least one subject hit a minimum heart rate of 30 beats per minute (BPM) or a maximum heart rate of 210 BPM at least once during the training period, as the Apple Watch heart rate sensor supports a range of 30-210 BPM [37]. Daily heart rates up to 210 BPM were recorded in most subjects during training days, and mean heart rates at night (between the hours of 8 PM and 4 AM), corresponding to sleep hours, were consistently 50% of mean rates during daytime hours. There were no significant associations between mean, range, daytime or nighttime heart rates in subjects who completed versus those not who did not complete training, or in categories of unsuccessful trainees (*P*>.05).

Table 4 compares the mean daily step count of all study subjects according to the completion category. Subjects who passed the course had higher mean daily step counts than those who did not (*P*<.05), and this was significant for all categories of noncompletion (DOR, *P*<.005; medical, *P*<.005; safety, *P*<.05; performance, *P*<.05). There were no significant differences in daily step counts between noncompletion categories.

Table 5 compares the mean active and resting energy expenditure (calorie) of the entire cohort according to the completion category. Subjects who passed the course had higher active energy expenditure than those who did not (*P*<.05), and this was significant for all categories except performance noncompletion (DOR, *P*<.001; medical, *P*<.05; safety, *P*<.05; performance, *P*<.06). There were no significant differences in daily step counts between noncompletion categories. Resting energy expenditure did not differ between those completing the course and completion failures, with the exception of DOR failures (*P*<.05).

**Table 3:** Mean heart rate (beats per minute) per group (course completion and all noncompletion categories) compared by time of day.

Group	24 hour		Day		Night	
	Mean (SD)	Range^a^	Mean (SD)	Range^a^	Mean (SD)	Range^a^
Pass (BPM^b^)	110 (30)	30-210	112 (29)	30-210	64 (16)	36-202
Drop on request (BPM)	106 (31)	30-210	111 (28)	30-210	64 (19)	40-171
Medical (BPM)	116 (29)	34-210	118 (28)	34-210	73 (19)	43-175
Safety (BPM)	111 (29)	30-208	113 (27)	30-208	69 (10)	41-139
Performance (BPM)	118 (28)	32-209	119 (27)	32-209	70 (13)	40-137

^a^Apple Watch heart rate sensor supports a range of 30-210 beats per minute [37].

^b^BPM: beats per minute.

**Table 4:** Mean activity (daily steps) per group (course completion and all noncompletion categories).

Group	Mean (SD)	Range
Pass (daily steps)	10,537 (3195)	500-15,311
Drop on request (daily steps)	4491 (4541)	239-15,057
Medical (daily steps)	4585 (4899)	456-15,537
Safety (daily steps)	6569 (4677)	780-11,787
Performance (daily steps)	7660 (3395)	2734-11,674

**Table 5:** Mean energy expenditure (calories) per group (course completion and all noncompletion categories) compared by activity level.

Group	Active^a^		Resting		
	Mean (SD)	Range		Mean (SD)	Range
Pass (calories)	1837 (1231)	1-9133		1523 (426)	107-4085
Drop on request (calories)	1329 (1266)	1-4911		1430 (496)	232-3851
Medical (calories)	961 (1294)	2-6021		1491 (527)	138-2652
Safety (calories)	1271 (1445)	1-5746		1410 (430)	133-1976
Performance (calories)	1450 (971)	11-3667		1413 (444)	207-1743

^a^Apple Watch uses logged workouts to calculate active energy expenditure.

### Survey Responses and Course Completion

The compliance of the baseline personality surveys was as follows: (1) BFI: 94.0% (108/115), (2) PANAS: 93.0% (107/115), (3) PPI: 76.5% (88/115), (4) SWLS: 92.2% (106/115), (5) ERS: 90.4% (104/115), (6) Grit: 88.7% (102/115), and (7) FFMQ: 83.5% (96/115). The baseline personality traits that were most associated with course completion include extroversion and positive affect.

Subjects who had DOR reasons for course failure versus those completing the course demonstrated an increase in emotional (*P*<.03) or physical pain (*P*<.002) and a degradation of confidence (*P=*.05), on a 1 to 5 grading scale, up to 3 days before withdrawal from the course.

There was no difference in self-reported feelings related to instructor support, adequacy of hydration, nutrition, or sleep duration between subjects completing or not completing the course. All trainees thought about quitting the course on at least one daily survey.

## Discussion

### Principal Findings

As the need for a modernized military grows, there is also a need to understand how best to involve larger numbers of warfighters in higher-level training and operations without degrading training and performance standards [2,4]. Here, we validated a model and platform yielding consistent and novel insights into successful warfighter training, the results of which can scale into other elite training and performance domains. We found novel physiological and psychological measurements capable of predicting success and failure in this difficult training course. Namely, the amount of physical energy expended early in training as well as positive affect and extraversion independently predict a lower probability of voluntarily leaving the course. These efforts represent a new model for research on military training and, perhaps most importantly, a validation of this approach for yielding insight past intuition regarding what constitutes high performance and fortitude in the face of training-related stressors.

Previous studies of elite military training have focused on psychological and physiological factors. In a previous study of Navy Sea, Air, and Land (SEAL) warfighters, compared with the general male population, SEALs scored lower in neuroticism and agreeableness and higher in extraversion and conscientiousness [38]. In a highly comprehensive review of factors pertinent for graduation from Basic Underwater Demolition/SEAL (BUD/S) training, Taylor et al [5] reviewed 13 studies of BUD/S candidates’ success rates. Across the studies reviewed, general themes revealed a range of physical fitness factors associated with graduation from BUD/S, although the results were not consistent across studies. Psychologically, emotional stability, adjustment, and likeability were implicated in individual reports but were also not consistently found to be predictive, highlighting the need for further inquiry that combines psychological and physiological measurements using modern devices. Prior studies have also identified that greater physical fitness predicts resilience in stressful environments [39] and that mental skills training can help ameliorate the deleterious effects of intense deployments on both mental state and stress hormones [40], together indicating the key interplay and joining of mental and physical functions. Although this study does not investigate all the potential cognitive and physiological interactions, our approach lays groundwork to bridge and unify these existing studies into natural settings where modern wearable technology can be leveraged in combination with physical and mental factors.

### Novel Insights From Direct and Indirect Measures of Trainee Physical Status

On the basis of this prospective study, we see evidence that the standard physical fitness tests and physiological measurements provide limited information regarding a Marine’s or Sailor’s likelihood of voluntarily dropping from training. Of all 4 land training standard assessments, only pull-ups were predictive of an increased probability of a DOR. Similarly, heart rate data was not a good predictor, perhaps reflecting the fact that trainees were young and healthy, and there was limited variability in the range of heart health among the sample, suggesting that their cardiovascular health was adequate to complete training.

The mean step counts of those who successfully completed the course were significantly higher than those who failed to complete the course, most of whom were removed from the course by training day 7. Not all training days involve physical training; subjects spent training days in a classroom environment as well, hence the wide range in mean daily step counts. Still, mean daily step counts correlate to approximately 2 to 3 miles daily in those who withdrew versus a mean of over 5 miles daily in those who completed the course. This signifies that most of the work output of the training course occurred after trainees withdrew, supporting the notion that they did not withdraw for physical reasons, and this may hint at broader clues as to the importance of motivation and personal narrative in determining success.

Indirect measures of energy expenditure, such as active energy, also indicate that those who successfully completed the course expended higher activity over time, versus those who were unsuccessful at completing the course. This was true for all categories of unsuccessful course completion except performance withdrawals. Those who were unsuccessful at completing the course due to performance withdrawal also tended toward higher step counts and were dropped due to failure to complete cognitive and physical tasks. This metric is also important because it encompasses and can measure training work done in water, which is not accounted for in step counts. The energy expenditure data are also revealing in that they provide the first comprehensive data on the total mean daily energy expenditure of trainees. Although the range is considerable, the observation that in the trainees who successfully completed the course approximately 3300 calories/day are needed to maintain caloric equilibrium is important knowledge in terms of nutritional planning and avoidance of weight loss [30,31]. These interfaces between psychological variables and energy expenditure stand to contribute greatly to models of performance, as the data collected over time can be used to design improvements to, and efficiency of, training schedule and requirements.

Although previous studies have examined the existing military training assessments, such as physical fitness scores [3], for the BRC, none have examined the individual tests that comprise the score (eg, pull-ups, sit-ups, hikes, and run) and none have examined BRPC. Furthermore, no other studies have collected longitudinal and continuous physiologic data and other wearable data from trainees during the entire duration of a training course.

### Personality and Emotional Status Assessment

We found that higher levels of self-reported positive affect and extraversion were significantly associated with successful course completion. Moreover, although all trainees entertained thoughts of quitting, those that did voluntarily or involuntarily withdraw were more introverted and had less demonstrative personality types than those that successfully completed the course. In contrast to previous research performed in military academies or in other military training [4,12-14], subject scores on the Grit Scale did not predict success or failure. However, the findings of this study are in line with other research that indicates a positive relationship between positive affect and psychological resilience in the US Military [41], sport performance [25,42], coping strategies in competitive athletes [43], and task performance [44]. Previous research also demonstrates a relationship between extraversion and military leadership [9] as well as sport performance [45,46]. A more detailed understanding of these differences is enabled by the daily survey data showing that students who DOR’d had a significant degradation in their self-reported emotional and physical pain and confidence scores, compared with those who successfully completed the course 1 to 2 days before withdrawal. This underscores the importance of daily and individualized assessment to gain fidelity into understanding exactly why or when a trainee may decide to quit. This new understanding points to a clear target for early intervention. Studies have shown that positive affect [47,48] and other mental skills training interventions [40,49,50] are feasible and effective; thus, future studies should determine if more positive affect and optimistic traits can be cultivated in these trainees to improve course completion rates and resiliency. As mentioned above, the ability to gauge in depth which training element (eg, aquatic events and overland hikes) responsible for the greatest amount of trainee doubt and anxiety offers the ability to direct resources toward building readiness for and confidence in completion of those training tasks.

### Validation of a New Model for Military Training Research

A unique aspect of this study is that it demonstrates the feasibility of a fully comprehensive and continuously collected model of human performance research in a naturalistic military training environment. We demonstrated the ability to collect accurate and continuous physical performance and psychological data throughout a 25-day course that takes place on land and in water. The success of the model was validated across the 3 successive training classes that we enrolled. The technology platform, Apple’s ResearchKit, HealthKit, and CareKit software *stack* coupled with the hardware components consisting of the iPhone and Watch provide important efficiencies and will further improve future studies through the integration of additional sensors and provide novel methods of data visualization in future apps [15-17]. The Apple technology stack was chosen for a few reasons. One reason is that biometric tracking on the Watch has been shown to be accurate and validated [18,19]. Another reason is that the cybersecurity and data privacy controls are state-of-the-art. In addition, we were able to leverage Apple’s ResearchKit to enroll subjects in the study through digital consent within the study app. Moreover, the devices were chosen because of subjects’ affinity for and familiarity with Apple products. The ResearchKit and HealthKit also provided the opportunity to bring in other survey data from the app and integrate Apple Watch and other data. Our app design process was collaborative and iterative. We regularly met with our software designers and the Reconnaissance Marines instructors as well as our research team to design and test versions of the app. The ResearchKit app enabled us to conduct a research study on an entirely digital platform, including the ability to consent subjects on their iPhones, and to custom design a Reconnaissance Marine app that served as the interface to the subject for collection of daily surveys.

Apple Watch data were collected using HealthKit, which provides the ability to collect Watch data and integrate it into the dataset collected on ResearchKit. In the future, we will leverage HealthKit to integrate other connected sensors such as weight, oxygen saturation, or temperature. The accuracy of the direct measures, such as heart rate, collected from the Watch using photoplethysmography, has been demonstrated by others to have a sensitivity and specificity of 98% and 90%, respectively [51]. As data in this study were continuously collected over the entirety of training days, we were also able to internally validate the heart rate measures by comparing daytime with nighttime readings, which were consistent with typical circadian variability of heart rates observed in a young, healthy male population [52]. The ability to perform research studies in a continuous model of data collection evolves the research model to one that is much more comprehensive and efficient than traditional models that focus on data collection during a single discrete study interval and are performed under more artificial and less naturalistic conditions than being embedded within the actual training environment being studied. It is particularly important to collect data for military populations while they perform their normal functions, which often include stressful conditions that are not easy to reproduce in a laboratory setting.

Other benefits of this model include the ability to more quickly and efficiently translate study findings into interventions that can be tested in the subsequent classes. For example, our finding that declines in both daily self-reported emotional and physical pain scores and confidence in graduating preceded DORs from training provide an opportune target to build and test interventions. In addition, the finding that most DORs occurred in relation to an aquatic training event suggests that these events are causing significant doubt, distress, and lack of confidence. The finding that individuals who completed the course manifest more positive affect suggests that an effective intervention should instill these traits and behaviors. Ideally, an intervention targeted at cultivating confidence for these activities will heighten the probability of successful completion without changing the rigorous nature of the training itself. This could include specially designed educational or motivational video content, which is delivered to the trainee manifesting these thoughts and feelings, through the next version of the study software app. Similarly, advances in technologies, such as the real-time electrocardiogram (ECG) capability on the Apple 4 Series Watch, can be easily integrated into the study and provide another opportunity to validate the heart rate data. These ECG and heart rate capture capabilities may provide a real-time safety component for these trainees, while undergoing intense physical training. For instance, if a trainee sustained a very elevated heart rate, this could be validated by obtaining an ECG from the Watch and displayed to instructors in real time, using a CareKit-enabled data dashboard, which could then allow for timely medical assessment of that individual.

### Future Studies

We have designed and implemented a flexible and continuous research model for military training that can easily accommodate best-in-breed sensors, allow for rapid software integration to more efficiently assess and test novel training interventions, and increase the course completion rate and quality of new Reconnaissance Marines and other military equivalents. This model can also provide important efficiencies and data flow that will help assess the medical well-being of trainees. We also found that despite the physical rigor involved in Reconnaissance Marine training, most of the attrition is due to mental deterioration rather than physical deterioration, and students who DOR often do so outside of the deepest physical stressors, that is, while on the pool deck as opposed to actually being in the pool. These signs of deterioration can be identified before trainees withdraw or are withdrawn from training, and interventions that target the reversal of these feelings as well as help to gain a deeper understanding of how to determine selection criteria can be designed.

### Limitations

We did not collect data on the entirety of the courses offered by the Reconnaissance Training Company, namely, the follow-on 60-day BRC that follows the 25-day BRPC, which limits our ability to draw conclusions about fully graduating into the Reconnaissance military occupational specialty. We also did not collect any data on weekends, mostly due to initial study design limitations with maintaining the study equipment inventory of iPhones, Watches, and data plans, which can be addressed through improved battery life and study design, leading to a model capable of measuring students during the entirety of trainee experience, including time off and personal time.

Daily questions (Multimedia Appendix 1) inquiring about substance use were only included in the second and third cohorts and inquired about nicotine and alcohol but not caffeine and other substances known to affect performance. In addition, the response scales were not standardized. For these reasons, the data were not analyzed and are a limitation in this study design. Future research should include a more comprehensive and standardized assessment of substances known to affect performance.

Although the Apple Watch has been validated in other studies [7,18,19,53], there were a number of possible limitations. Although Apple does not promote a specific sleep analysis feature, heart rate data have been known to correlate with sleep patterns, and recent research has begun to validate the use of raw Apple Watch heart rate data to derive sleep duration [54]. One limitation was the range of heart rates that the Apple Watch is technically able to collect, which consists of a minimum of 30 BPM and a maximum of 210 BPM, as highlighted in Table 3 [37]. For instance, an individual could register a heart rate of 30 BPM, such as a physiological pause after a premature beat. Similarly, a high heart rate recorded at 210 BPM would not necessarily mean that the heart rate was sustained at this level but that it was sustained enough to register at the upper limit. The frequency of low or high heart rate events depends on the frequency of sampling at a higher rate in workout mode (every 5 seconds) versus regular mode (every 5 min). Therefore, the mean heart rates are more representative of our cohort’s data. The fact that we reported within our range values both very low and very high step counts and energy expenditures is a result of a number of factors. If a trainee was removed from training early, spent the day in a classroom, or did not enter the workout mode, this would account for low step count and energy expenditure. For these reasons, we feel that the mean values are more reflective of the data. However, to control for these limitations, an additional and different wearable sensor with a wider range of data collection capabilities can be used in future studies in conjunction with the Apple Watch to further compare data.

Owing to the nature of military training, another limitation is that the number of subjects per group is highly unequal. To address this, we combined data from the 3 cohorts across groups to create larger sample sizes. In the future, we will require additional cohorts to increase the sample size and validate the results.

Another possible limitation of our study is the use of self-report surveys, which can be prone to bias or misreporting. However, other studies in military populations have used the same or similar personality and behavioral self-report surveys [8,11-13,15,23,25,26]. Although we agree that self-assessment is not generalizable, our ability to obtain daily individual measures of mental and behavioral status, we feel, was a great strength in this study. Furthermore, we recognize that all observational research has the potential for bias to some degree, but we feel as though this research is crucial in contributing to human performance knowledge by not disrupting the natural training environment of these elite warfighters.

### Conclusions

This study demonstrates the feasibility and accuracy of actionable data that can be collected using a natural, continuous, and holistic model of data collection using a modern digital platform, custom-made software, and body-worn sensors. This model of innovation has the potential for rapid discovery in military training environments that may lead to better training and selection of military personnel and translate into other elite training environments. In addition, the data paint a novel picture of the mind and body processes in determining performance outcomes—findings that can generalize into any high pressure, competitive domain.

## Appendix

Multimedia Appendix 1 Daily survey questions.

Multimedia Appendix 2 Rates of successful course completion and failure to complete by category for all 3 classes. DOR: drop on request.

## Abbreviations

BFI: Big Five Inventory
BPM: beats per minute
BRC: Basic Reconnaissance Course
BRPC: Basic Reconnaissance Primer Course
BUD/S: Basic Underwater Demolition/SEAL
DOR: drop on request
ECG: electrocardiogram
ERS: Ego Resilience Scale
FFMQ: Five Facet Mindfulness Questionnaire
PANAS: Positive and Negative Affect Scale
SEAL: Sea, Air, and Land
SOI-W: School of Infantry-West
SWLS: Satisfaction with Life Scale
