# Supplementary material for: Continuous Measurement of Reconnaissance Marines in Training With Custom Smartphone App and Watch: Observational Cohort Study
Source: JMIR Mhealth Uhealth. 2020 Jun 15;8(6):e14116. doi: 10.2196/14116 (PMC7324996; doi:10.2196/14116)
Supplement: Multimedia Appendix 1 [file mhealth_v8i6e14116_app1.docx]

**Appendix A:**

| **Daily Survey Questions** | |
| --- | --- |
| How would you rate your physical pain (i.e. joint)? | 1=No pain : 5=Worst pain imaginable |
| How do you feel right now? | 1=Terrible : 5=Great |
| Did you feel that you had enough food to eat today? | TRUE/FALSE |
| Approximately how many liters of fluid did you drink today? | 1 – 10 |
| Approximately how many hours did you sleep last night? | 0 – 12 |
| My trainer’s feedback helped me perform better today. | 1=Very Much : 5=Not at All |
| How confident are you right now that you will graduate? | 1=I don’t think I’m going to make it, but I will keep going until they drop me : 5=Completely confident that I will graduate |
| Did I consider quitting at any point today? | TRUE/FALSE |
| Do I think I will quit before this training is complete? | TRUE/FALSE |
| In the past 24 hours, have you consumed any alcoholic beverages? | YES/NO |
| *If Yes, how many drinks did you consume in the last 24 hours?* | *N/A, 1 - 10, > 10* |
| In the past 24 hours, have you used tobacco products? | YES/NO |
| *If you do use tobacco products, how long does a pack, can, etc. last?* | *N/A, 1 day - 7 days, > 1 week* |
