# Supplementary figures and images for: Continuous Measurement of Reconnaissance Marines in Training With Custom Smartphone App and Watch: Observational Cohort Study
Source: JMIR Mhealth Uhealth. 2020 Jun 15;8(6):e14116. doi: 10.2196/14116 (PMC7324996; doi:10.2196/14116)

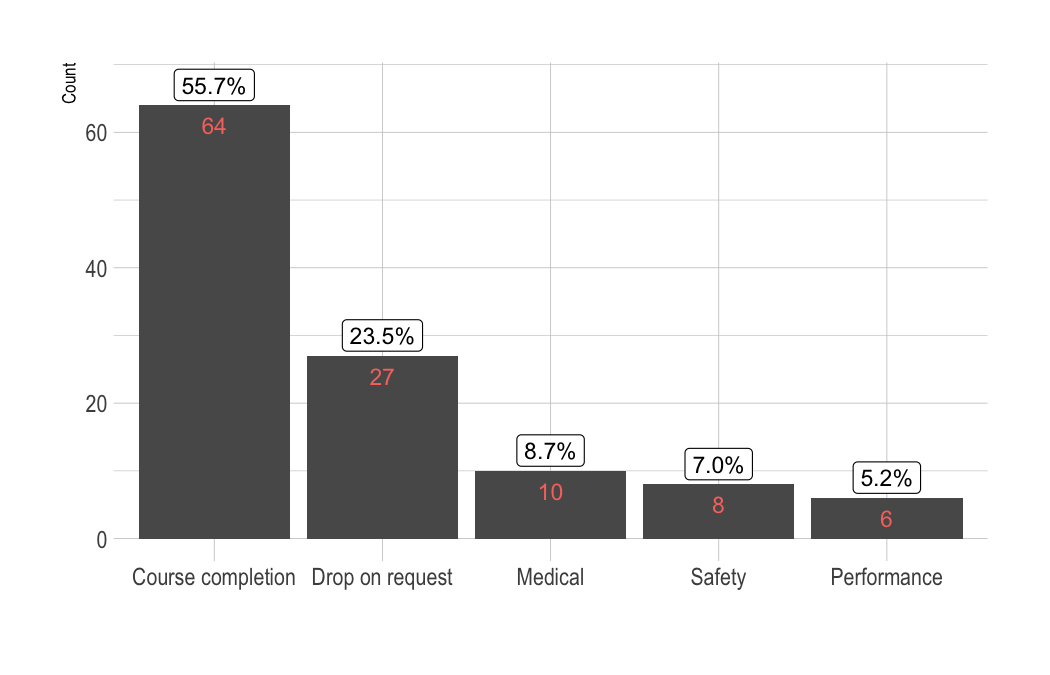

Supplement: Multimedia Appendix 2 [file mhealth_v8i6e14116_app2.png]
